# Alterations in Monocyte CD16 in Association with Diabetes Complications

**DOI:** 10.1155/2012/649083

**Published:** 2012-12-18

**Authors:** Danqing Min, Belinda Brooks, Jencia Wong, Robert Salomon, Wensheng Bao, Brian Harrisberg, Stephen M. Twigg, Dennis K. Yue, Susan V. McLennan

**Affiliations:** ^1^Department of Endocrinology, Royal Prince Alfred Hospital, Sydney, NSW 2050, Australia; ^2^Discipline of Medicine and Bosch Institute, The University of Sydney, Sydney, NSW 2006, Australia; ^3^Diabetes Centre, Royal Prince Alfred Hospital, Sydney, NSW 2050, Australia; ^4^Sydney Nursing School, The University of Sydney, Sydney, NSW 2006, Australia; ^5^Department of Ophthalmology, Royal Prince Alfred Hospital, Sydney, NSW 2050, Australia

## Abstract

Monocytes express many cell surface markers indicative of their inflammatory and activation status. Whether these markers are affected by diabetes and its complications is not known and was investigated in this study. Blood was obtained from 22 nondiabetic and 43 diabetic subjects with a duration of diabetes >10 years, including 25 without and 18 with clinically significant complications. The number of CD45^+^CD14^+^ monocytes and the percentage expressing the proinflammatory marker CD16 were determined by flow cytometry. Other markers of monocyte activation and expression of chemokine receptors were also examined. The relationship between monocyte CD16 and clinical data, selected cytokines, and chemokines was also investigated. Diabetes had no effect on total white cell number but increased monocyte number. Diabetes also significantly decreased the number of CD16^+^ monocytes but only in those with diabetic complications. Other markers of monocyte activation status and chemokine receptors were not affected by diabetes or complications status. Diabetes induced plasma proinflammatory cytokines and they were lower in diabetic subjects with complications compared to those without complications. These results suggest that the circulating monocyte phenotype is altered by diabetic complications status. These changes may be causally related to and could potentially be used to predict susceptibility to diabetic complications.

## 1. Introduction

The complications of diabetes are responsible for much of its associated morbidity and mortality. Landmark studies have shown that the risk for development of diabetic complications increases with poor glycemic control and disease duration [1–3], although other factors such as inflammation are also likely to be important [4–7]. The levels of circulating proinflammatory cytokines and chemokines such as tumor necrosis factor-*α* (TNF-*α*), interleukin-1*β* (IL-1*β*), and monocyte chemoattractant protein-1 (MCP-1) are commonly used as markers of inflammation, and they have been shown to be increased in diabetes [8–10]. Whether monocytes, a cell type central to the inflammatory response, are affected by the diabetic milieu and provide complementary information is not known.

Circulating monocytes are identified by flow cytometry as CD45^+^CD14^+^ cells. They are further characterized according to the presence or absence of expression of CD16, the low-affinity Fc receptor, Fc*γ*RIII [11]. The CD16^−^ monocytes constitute a “classically activated” subset of monocytes which account for 80–90% of circulating monocytes in normal healthy individuals. This population is increased in acute inflammation and is rapidly recruited to sites of infection [11–13]. The CD16^+^ monocytes constitute the “nonclassically activated” subset which makes up the remainder of the monocyte population. This CD16^+^ population has a patrolling function to sense tissue injury, and is increased in ageing and chronic inflammatory disorders [11–16]. In acute conditions, these subsets express different levels of chemokine receptors, the CD16^−^ subset expresses CCR2 (chemokine receptor 2), the receptor for MCP-1, and the CD16^+^ subsets expresses CCR5 (chemokine receptor 5) which binds macrophage inflammatory protein-1*β* (MIP-1*β*) [11, 17]. The CD16^+^ subset typically responds to bacterial endotoxin by secretion of proinflammatory cytokines, in particular TNF-*α* and IL-1 [11, 15, 18]. Monocytes also express other cell surface proteins which can provide information regarding their inflammatory activation status. For example, in response to inflammatory stimuli 27E10 is expressed in the acute phase whilst 25F9 is increased at later stages [19–21]. Additionally, CD68 is a marker for tissue macrophages, and CD11b is expressed by activated monocytes [6, 22, 23]. 

Diabetes has been shown to alter the circulating monocyte populations [24–26], but the relationship between these changes and the presence of diabetic complications has not been investigated. In the current study, we used flow cytometry to examine the circulating monocytes in particular CD16^+^ monocytes and other markers of monocyte activation such as 27E10, 25F9, CD68, and CD11b, in two groups of diabetic subjects with an equally long duration of diabetes but which differ by the presence or absence of clinically significant diabetic complications. Results were compared with nondiabetic control subjects. The relationship between the expression of these markers and circulating proinflammatory cytokines and chemokines was also investigated.

## 2. Materials and Methods

### 2.1. Study Participants

Patients with no micro- or macrovascular complications (D^−Comps^), despite a duration of diabetes greater than ten years, were identified from the clinical database of the Diabetes Centre, Royal Prince Alfred Hospital and Central Sydney Eye Surgeons. Patients with similarly long durations of diabetes, but with diabetic complications (D^+Comps^), were also recruited from the same database. In total, 43 patients (24 males and 19 females; mean age 62.7 ± 11.7 years) with type 1 (*n* = 7) and type 2 (*n* = 36) diabetes were studied. Based on standard screening, 18 subjects with complications and 25 without clinically significant microvascular and/or macrovascular disease were recruited. The presence of retinopathy was confirmed by fundal examination and/or photography. The absence of diabetic nephropathy was confirmed by normal serum creatinine and urinary albumin/creatinine ratio (U Alb/Cr) <2.5 mg/mmol for males and <3.5 mg/mmol for females. Study participants were considered to have macrovascular disease if they had any relevant symptoms of vascular disease or had reported a history of abnormal investigation or prior macrovascular event. In addition, a total of 22 nondiabetic participants (6 males and 16 females; age 49.7 ± 9.0 years) were recruited as controls from the general community or staff members of the hospital. The study has the approval of the Ethics Review Committee of the institution and was carried out in accordance with the principles of the Declaration of Helsinki as revised in 2000. All study participants gave their written informed consent.

### 2.2. Multicolor Flow Cytometry

Multicolor flow cytometry was used to characterize the monocyte subsets under investigation, and the following monoclonal antibodies conjugated with the fluorochromes fluorescein isothiocyanate (FITC), phycoerythrin (PE), allophycocyanin (APC), peridinin chlorophyll protein-cyanine5.5 (PerCP-Cy5.5), and allophycocyanin-cyanine7 (APC-Cy7) were used. They are anti-CD45 (PerCP-Cy5.5 and APC), anti-CD14 (FITC and PerCP-Cy5.5), anti-CD16 (PE), anti-CD68 (PE), anti-27E10 (PE), anti-25F9 (FITC), anti-CD11b (APC-Cy7 and PE), anti-CCR2 (APC), and anti-CCR5 (APC). Appropriate mouse IgG subclasses (FITC, PE, APC, PerCP-Cy5.5, and APC-Cy7) were used as negative controls. The antibodies were obtained from Becton Dickinson (BD, San Jose, CA), R&D Systems (Minneapolis, MN, USA), Abcam (Cambridge, UK), and Santa Cruz Biotechnology (Santa Cruz, CA, USA), respectively. 

For analysis, venous blood was collected into EDTA-treated tubes. Whole blood (100 *μ*L) was then incubated with fluorescent-conjugated monoclonal antibodies to CD45 and CD14 in combination with a fluorescent conjugated antibody to CD16, 27E10, 25F9, CD68, CD11b, CCR2, or CCR5. After a 60-minute incubation at 4°C, the erythrocytes were lysed by the addition of 0.4% saponin (Sigma, Australia). The cells were washed immediately with Phosphate Buffered Saline (PBS) and collected by centrifugation at 300 g for 5 minutes. The supernatant was discarded, and the cell pellet was resuspended and washed in FACS buffer (PBS containing 0.5% w/v BSA, 0.1% w/v NaN_3_, and 2 mM EDTA). Cell surface marker expression was then determined by flow cytometry using either the BD FACSCanto or BD FACSAria (Becton Dickinson, San Jose, CA, USA). A minimum of 30,000 events were counted for each CD marker set. All samples were analyzed in parallel. Conjugated isotype controls, single antibody-stained controls, and fluorescence-minus-one (FMO) controls were also included.

**Figure 1:**
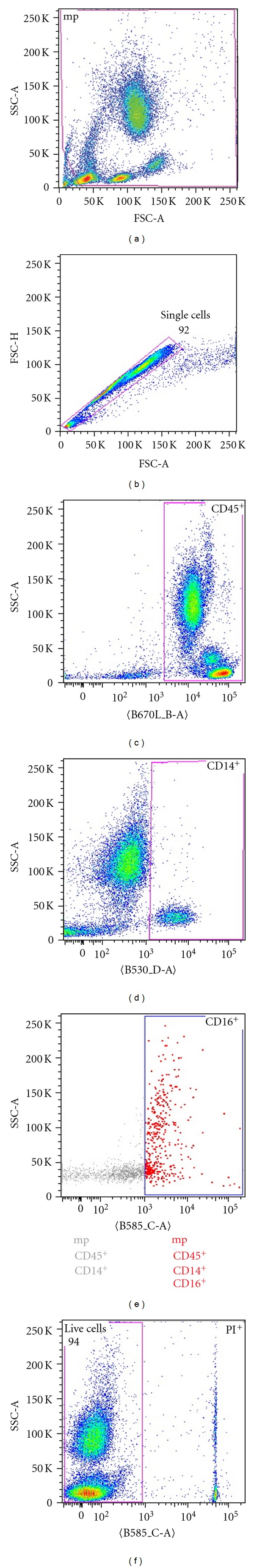
Flow cytometry gating strategy. Flow cytometry data obtained from a representative sample is shown. (a) Peripheral blood cells were first gated based on forward scatter (FSC-A)/side scatter (SSC-A), with exclusion of dead cells. (b) The events were then visualized using FSC-A/FSC-H dot plot, and the singlets (single cells) were gated. (c) Leukocytes were identified by their positive staining with CD45. (d) Monocytes were then defined as the CD14^+^ cells within the CD45^+^ leukocyte population. (e) The final gating was based on the CD marker of interest within the CD45^+^CD14^+^ cell population. (f) Necrotic cells in the whole blood sample were detected by Propidium Iodide (PI) staining.

The data was analyzed using FlowJo version 8.1.1 (Ashland, OR, USA). Leukocytes were initially identified and separated into subgroups based on cell size (forward scatter) and granularity (side scatter) with dead cells excluded. The sequential gating strategy is shown in detail in Figure 1. The primary gating focused on the CD45^+^ population, the secondary gating on the CD14^+^ cells, and the final gating was based on the CD^+^ marker of interest within the CD45^+^CD14^+^ cell population. The results were then expressed as the percentage of CD^+^ cells within the CD45^+^CD14^+^ population.

### 2.3. Measurement of Plasma Cytokine Levels

The circulating concentrations of proinflammatory cytokines and chemokines IL-6, IL-8, TNF-*α*, interferon-gamma (IFN-*γ*), interferon gamma-induced protein 10 kDa (IP-10), MCP-1, and MIP-1*β* were measured in plasma using a Bio-Plex Pro Assay (Bio-Rad, Hercules, CA, USA). The concentration of IL-10 was quantified using a commercially available ELISA kit from R&D Systems (Minneapolis, MN, USA) according to the manufacturer's instructions.

### 2.4. Statistical Methods

Data analysis was performed using the NCSS 2004 statistical package. Continuous data were checked for normality and are presented as mean ± standard deviation or median and interquartile range. Grouped data were compared by *t*-test. Linear regression and correlation analysis was used to verify the significance of the relationship between monocyte subsets and the concentrations of proinflammatory cytokines, chemokines, and clinical variables, respectively. Statistical significance was accepted at *P* < 0.05. 

## 3. Results

### 3.1. Clinical Profile

Demographic and clinical parameters of study participants are shown in Table 1 (individual patient data is shown in Supplementary Table 1  available online at doi:10.1155/2012/649083). The age of the control subjects ranged between 34.6 and 65.4 years whilst the diabetes' group ranged between 30.7 and 78.8 years. The groups of diabetic patients were well matched for diabetes type (D^−Comps^: *n* = 3 T1DM versus D^+Comps^: *n* = 4 T1DM), age (D^−Comps^: 63.3 ± 8.6 versus D^+Comps^: 61.8 ± 15.2), and duration of diabetes (D^−Comps^: 17.8 ± 6.7 versus D^+Comps^: 21.8 ± 8.1). The group with complications comprised subjects with microvascular disease alone (*n* = 14) or both micro- and macrovascular disease (*n* = 4) and had slightly poorer glycemic control and worse renal parameters. Between the groups, there were no significant differences in treatment with statins (D^−Comps^: 21/25, D^+Comps^: 14/18), antihypertensive agents (D^−Comps^: 19/25, D^+Comps^: 15/18), or aspirin (D^−Comps^: 11/25, D^+Comps^: 12/18).

### 3.2. The Effects of Diabetes on Monocyte Number and Morphology

**Figure 2:**
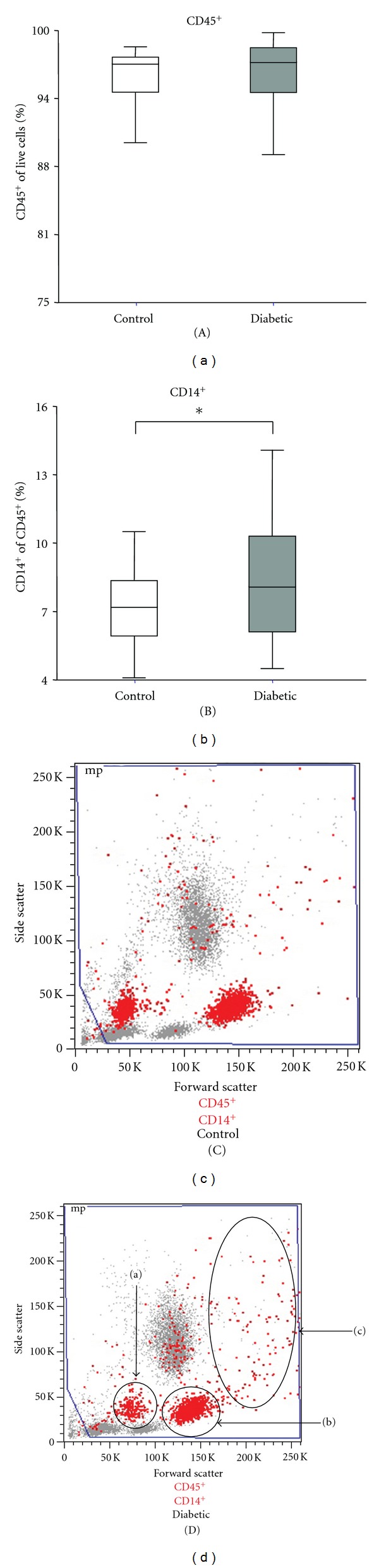
The effect of diabetes on circulating monocytes. Leukocytes and monocytes were identified by flow cytometry. Shown in control and diabetic subjects are (A) the CD45^+^ leukocyte population and (B) the CD45^+^CD14^+^ monocyte population. (C) Representative forward scatter and side scatter plots from a control and diabetic subject showing CD45^+^CD14^+^ monocytes (red dots). The three subsets of monocytes grouped according to size and granularity are shown in the diabetic sample as (a), (b), and (c). *P < 0.05 different between groups.

There was no effect of diabetes on the number of circulating white cells (CD45^+^ cells), but the monocyte component (CD45^+^CD14^+^) was significantly increased (diabetic: 8.3 ± 2.6% versus control: 7.2 ± 1.6%, *P* < 0.05) (Figures 2(A) and 2(B), resp.). This increase was not affected by diabetes type (T1DM: 7.2 ± 2.4% versus T2DM: 8.5 ± 2.6%, *P* = 0.22) or complications status (D^−Comps^: 8.2 ± 2.7% versus D^+Comps^: 8.5 ± 2.5%, *P* = 0.72). As shown in the representative flow cytometry scatter plots from control and diabetic subjects (Figure 2(C)), the monocytes were morphologically heterogeneous, showing a wide scatter in size (forward scatter) and granularity (side scatter). Based on these characteristics, three populations were identified as a smaller size and less granular population (a), an intermediate population (b), and a population of monocytes which is larger in size and more granular in group (c). These larger more granular monocytes (group (c)) were responsible for the 1% increase in monocyte number seen in diabetes (diabetic: 3.8 ± 2.3% versus control: 2.7 ± 1.4%, *P* < 0.05).

### 3.3. The Effects of Diabetes and Complications Status on Monocyte CD16 Expression

**Figure 3:**
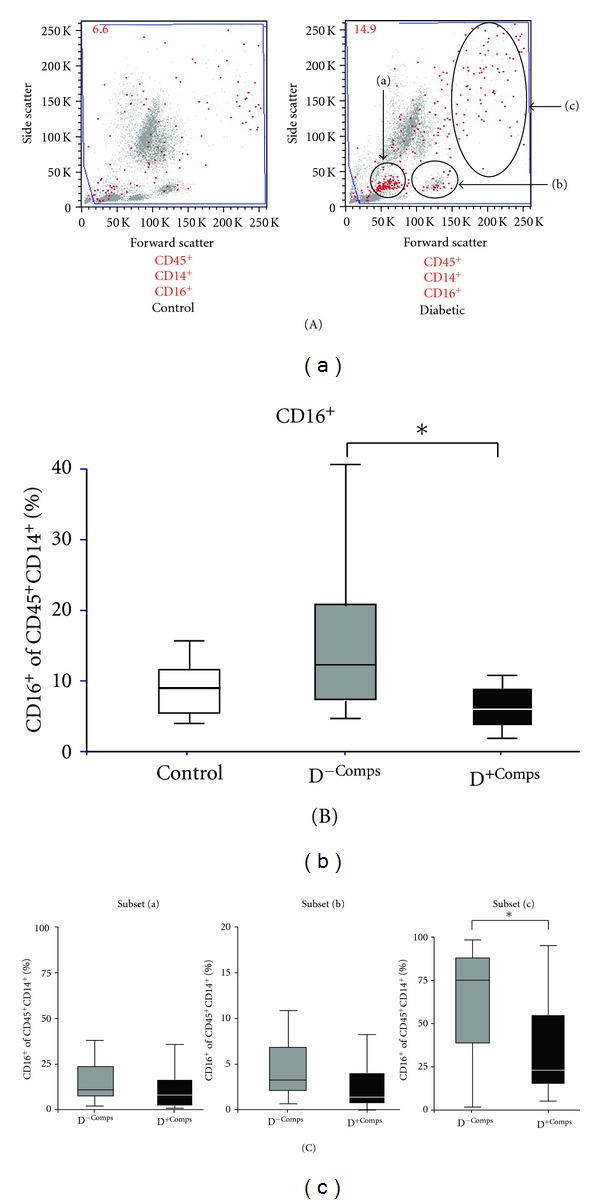
The effect of diabetes and its complications on percentage of CD16 monocytes. (A) Representative forward scatter and side scatter plots data from a control and diabetic subject showing the CD16^+^ cells (red dots) within the CD45^+^CD14^+^ monocyte population. (B) The percentage of CD16^+^ monocytes in control and diabetic subjects with and without complications. (C) The percentage of CD16^+^ monocytes in each monocyte subset grouped according to size and granularity. *P < 0.05 different between groups.

Representative flow cytometry plots of CD45^+^CD14^+^CD16^+^ monocytes from a nondiabetic and a diabetic subject are shown in Figure 3(A). Overall, there was no significant difference in the percentage CD16^+^ monocytes between nondiabetic control and diabetic subjects (control: 8.5 ± 3.4% versus diabetic: 11.4 ± 8.8%). However, within the diabetic cohort, the percentage of CD16^+^ monocytes was lower in those subjects with diabetic complications compared to those without complications (Figure 3(B)). This result was also seen when only those with T2DM were analyzed (D^−Comps^: 14.9 ± 9.4% versus D^+Comps^: 8.2 ± 7.5%, *P* < 0.05). The increase in the percentage of CD16^+^ cells observed in the diabetic cohort without complications was substantially due to an increase in the larger size and more granular monocytes (population (c) in Figure 3(C)). Regression analysis showed a relationship between cholesterol and CD16 expression (*r* = 0.306,   *P* = 0.05) accounting for approximately 9.3% of the variance. No such relationship was observed for HbA1c.

### 3.4. The Effects of Diabetes on Other Monocyte Markers of Inflammation and Differentiation

**Figure 4:**
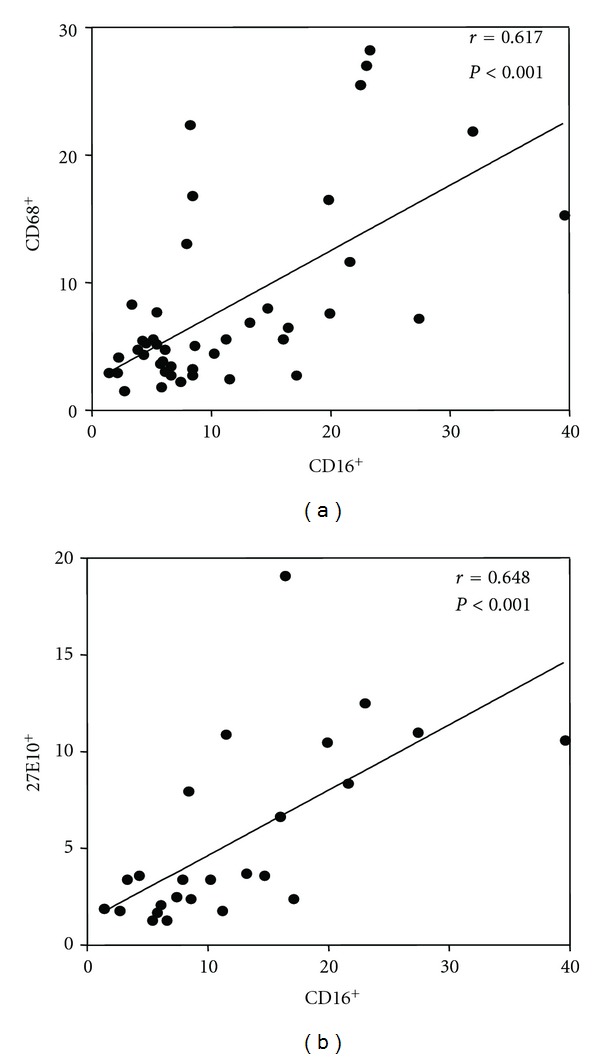
The relationship between CD16^+^ monocyte and other CD markers. The relationship between the CD16^+^ monocyte and (a) macrophage marker CD68^+^ and (b) 27E10^+^, a marker of acute inflammatory response.

Diabetes and its complications status had no effect on monocyte expression of chemokine receptors CCR2 and CCR5, or any of the differentiation markers 27E10, 25F9, CD68, and CD11b. Shown in Figure 4, there were significant correlations between the percentage of CD16^+^ monocytes and the percentage of monocytes expressing the differentiation markers CD68^+^ and 27E10^+^.

### 3.5. Plasma Levels of Inflammatory Cytokines and Chemokines

The plasma concentration of cytokines and chemokines in the control and diabetic subjects are shown in Table 2. Diabetic patients with complications had lower levels of the proinflammatory cytokines IL-6, IL-8, TNF-*α*, and IFN-*γ* but a higher level of the anti-inflammatory cytokine IL-10 when compared with their counterparts without complications. By contrast the complications group had higher chemokine levels with MIP-1*β* reaching statistical significance. No correlation between percentage of CD16^+^ monocytes and circulating inflammatory cytokines and chemokines was observed.

## 4. Discussion

Inflammation plays a central role in the development of diabetic complications, and macrophages in the tissue are known to be an important cell type in this regard [5, 27, 28]. However, studies on macrophages are hampered by the difficulty of obtaining appropriate tissue samples. In this regard, circulating monocytes may provide a valuable alternative source of material to test various hypotheses. Monocytes are derived from myelomonocytic stem cells in the bone marrow where they mature to monocytes. Once in the blood, they develop further and migrate in response to chemokines to the tissue where they differentiate into functionally and phenotypically distinct macrophage types. This heterogeneity of tissue macrophages is well established. Their phenotype and function are known to be altered in response to multiple factors, including bacterial endotoxins, tissue injury and inflammatory signals [11, 29]. On the other hand, little is known regarding monocyte heterogeneity in diabetes. Further, whether any changes reflect clinical and complication status of this condition has not been studied. In this study, we used flow cytometry to identify and examine the expression of a variety of cell surface markers on monocytes obtained from peripheral blood.

Similar to the study of Corrales et al., we observed an increase in circulating monocytes in diabetic subjects compared with nondiabetic controls [30]. It is of interest that the increase can be substantially attributed to the population with larger cell size and higher granularity (subset (c)), phenotypic features more like that of a macrophage. When analyzed as a single cohort, there was little difference between normal and diabetic monocyte expression of CD16. However, this masked the higher expression of these markers in the diabetic cohort with no complications despite a long duration of diabetes. These results are similar to those published by Mysliwska et al. which showed increased CD16^+^ monocytes in young T1DM subjects without retinopathy compared to those with retinopathy [31]. Unlike our study, this study showed an increase in the CD16^+^ monocytes in the diabetic cohort compared with nondiabetic controls. Why this difference occurs is not certain, but factors such as age and BMI of the cohort studied are likely to be important [24]. Interestingly, in our study, the main difference in the monocyte morphology appeared to be due to shape of the monocyte with the biggest changes being in the larger and more granular monocytes. Perhaps due to related mechanisms, there were strong correlations between CD16 expression and CD68^+^ and 27E10^+^, both markers of monocyte activation and differentiation. 

The results of plasma cytokines and chemokines showed some interesting parallels with the monocyte cell surface marker studies. The diabetic subjects with no complications again were different from those with complications in that they have higher proinflammatory cytokine levels. However, IL-10, known to be more anti-inflammatory in action, did not follow this trend. Whilst it is not possible to distinguish the monocyte subset contributing to the circulating cytokine levels, this increase maybe due to the increased percentage of IL-10 secreting CD16^−^ monocytes in those diabetic subjects with complications [25]. This finding of higher proinflammatory cytokine levels in those without complications was somewhat unexpected. There are variable reports regarding proinflammatory cytokine levels and their association with diabetic complications [32–34]. Most studies have focused on the presence or absence of cardiovascular disease or diabetic nephropathy [32–34]. Our diabetic subjects had long durations of disease and were mostly T2DM with the majority having diabetic retinopathy in the earlier stages (only one with proliferative disease). The data regarding proinflammatory cytokine levels in subjects with retinopathy is less clear. There are few studies, and both increased and decreased levels have been described [31, 35–37]. For example, in T1DM subjects with nonproliferative diabetic retinopathy, TNF-*α* has been reported to be higher than the level seen in subjects without diabetic retinopathy [31]. In contrast, in a T2DM cohort, TNF-*α* level was shown to change depending on the stage of disease. In their study, the decreased TNF-*α* and IL-6 levels were observed in the subjects with nonproliferative diabetic retinopathy, whilst the presence of proliferative diabetic retinopathy was associated with the increased TNF-*α* and IL-6 levels [35]. How factors such as complications type and severity, disease duration, age, and type of diabetes affect proinflammatory cytokine levels remains to be systematically studied. 

 Together these studies of monocytes and plasma suggest that pro- and anti-inflammatory markers are altered in diabetes in a manner reflecting different status of diabetic complications. As mentioned somewhat counterintuitively, compared to those with complications, those diabetic subjects with no complications have higher circulating proinflammatory markers (CD16^+^, IL-6, IL-8, TNF-*α*, and IFN-*γ*). Despite this pattern, we cannot conclude directly that the monocytes are overall more anti-inflammatory because the relationship between function and surface marker expression may not be a proportional one. It also remains to be proven in animal studies that the change in the profile of circulating monocytes in diabetes observed in the present study has any relationship to those occurring in tissue macrophages. In addition, there are other monocyte subsets and surface markers associated with different inflammatory states which remain to be studied. For example, a recent report by Gonzalez et al. has shown that high glucose concentration can downregulate CD33, a membrane receptor, but increase secretion of monocyte proinflammatory cytokines [26]. However, the expression of this receptor in diabetic subjects with complications was not examined. Additionally, to assess the relationship between the percentage of monocytes expressing the surface marker and the net inflammatory status of monocytes, functional studies would need to be performed on purified monocytes and on their various subsets. The observed pattern of cells of different size and granularity suggests altered activation status of the monocytes and adds another level of complexity which may need to be studied separately. In addition, it is known that *in vivo*, a large pool of monocytes adheres to the endothelial cell surface [38]. Our results showed that diabetic subjects with complications may have higher chemokine levels. As such, their pool of adhered monocytes may be greater and differently constituted. This may also have a bearing on the results of experiments. 

How the changes we have observed are mediated mechanistically needs to be answered with further investigations. Whether the changes of monocyte surface markers with diabetic complications is a causal one cannot be determined from this study. The slight differences in glycemic control and renal function between the two groups of diabetic subjects do not seem to play a significant role. In animal studies, some of these questions can be answered by observing the effects of suppressing individual subtypes of monocytes. In human studies, further insight can be obtained by monitoring longitudinally monocyte and plasma markers early in the natural history of diabetes before development of diabetic complications. Modalities of treatment proven to impart benefit on the microvascular or macrovascular complications (e.g., treatment with statin) can also be evaluated in this setting. Our diabetic cohorts are selected from a background of longstanding diabetes according to the presence or absence of complications using a composite criteria and assessed comprehensively at our Diabetes Complications Assessment Service [39]. Although the cohorts studied are heterogeneous, the grouping used in our study represents patterns commonly seen clinically but which cannot be readily explained. Future studies could further examine each complication separately. However, clinical complications tend to coexist, and it would be difficult to recruit individuals affected by only one type of complication. Although many questions remain unanswered, our study opens the possibility that peripheral blood monocytes can be used to investigate the pathophysiology of diabetic complications.

## Supplementary Material

Supplemental Table 1: listed the details regarding individual complications status of all diabetic subjects in this study

## Figures and Tables

**Table 1:** Demographic and clinical parameters of control and diabetic subjects with or without complications.

	Control	Diabetic	Diabetic	
	(*n* = 22)	(*n* = 43)	D^−Comps^ (*n* = 25)	D^+Comps^ (*n* = 18)
Age (yrs)	49.7 ± 9.0	62.7 ± 11.7*	63.3 ± 8.6	61.8 ± 15.2
Duration (yrs)	n.a.	19.5 ± 7.5	17.8 ± 6.7	21.8 ± 8.1
Weight (kg)	76.3 ± 18.7	79.9 ± 14.1	79.3 ± 14.8	80.7 ± 13.5
BMI (kg/m^2^)	28.5 ± 7.8	28.7 ± 4.9	29.1 ± 5.1	28.2 ± 4.7
HbA_1c_ (%)	n.a.	7.5 ± 1.1	7.1 ± 0.6	8.2 ± 1.2^†^
Serum creatinine (*μ*mol/L)^#^	66.0 (62.3–80.0)	80.0 (70.0–95.0)*	70.0 (63.5–83.5)	89.5 (79.8–103.8)^†^
U Alb/Cr ratio (mg/mmol)^ #^	0.7 (0.6–1.2)	1.5 (0.8–3.0)*	0.9 (0.6–1.5)	3.2 (1.9–8.9)^†^
eGFR (mL/min)	91.4 ± 16.9	80.8 ± 24.4	86.6 ± 23.0	72.6 ± 24.6
Triglycerides (mmol/L)	1.2 ± 0.5	1.5 ± 0.8	1.6 ± 0.8	1.5 ± 0.8
HDL cholesterol (mmol/L)	1.8 ± 0.5	1.3 ± 0.4*	1.4 ± 0.3	1.2 ± 0.4
LDL cholesterol (mmol/L)	3.0 ± 0.6	2.1 ± 0.7*	2.2 ± 0.8	1.9 ± 0.6
Sys BP (mmHg)	116 ± 14	127 ± 16*	124 ± 16	132 ± 15
Dias BP (mmHg)	77 ± 8	74 ± 7	74 ± 7	74 ± 7

Results are expressed as mean ± SD, except (^#^) shown as median with interquartile range (IQR).

Significantly different if *P* < 0.05 indicated as (*) for controls versus diabetes and (^†^) for D^−Comps^ versus D^+Comps^.

**Table 2:** Plasma levels of cytokines and chemokines in control and diabetic subjects.

(pg/mL)		Control	Diabetic	Diabetic	
		(*n* = 22)	(*n* = 43)	D^−Comps^ (*n* = 25)	D^+Comps^ (*n* = 18)
Cytokines	IL-6^#^	3.2 (2.1–5.3)	3.8 (2.7–4.8)	4.5 (3.2–7.1)	3.6 (2.6–4.2)
	IL-8^#^	3.1 (1.8–4.4)	4.5 (3.3–5.5)*	5.1 (3.7–6.4)	3.7 (2.9–4.6)^ †^
	TNF-*α* ^#^	10.4 (7.6–20.1)	12.2 (8.5–19.8)	12.8 (10.4–23.8)	10.0 (5.5–12.8)^ †^
	IFN-*γ* ^#^	61.6 (46.6–85.5)	56.8 (49.3–76.9)	68.4 (53.5–113.4)	50.3 (45.6–59.7)^ †^
	IL-10^#^	0.03 (0.00–0.43)	0.52 (0.07–0.94)*	0.46 (0.00–0.82)	0.83 (0.20–1.08)

Chemokines	IP-10	449.4 ± 134.6	483.7 ± 161.4	463.4 ± 159.2	511.9 ± 164.7
	MCP-1^#^	14.7 (12.0–22.1)	21.7 (15.0–35.7)*	19.2 (14.8–35.1)	23.6 (15.7–43.4)
	MIP-1*β*	20.4 ± 8.8	23.7 ± 12.4	20.3 ± 11.0	28.5 ± 12.9^†^

Results are expressed as mean ± SD, except (^#^) shown as median with interquartile range (IQR).

Significantly different if *P* < 0.05 indicated as (*) for controls versus diabetes and (^†^) for D^−Comps^ versus D^+Comps^.

## Abbreviations

CCR: C-C Chemokine receptor
IP-10: Interferon gamma-induced protein 10 kDa
MCP-1: Monocyte chemoattractant protein-1
MIP-1*β*: Macrophage inflammatory protein-1*β*
U Alb: Urinary albumin
U Alb/Cr: Urinary albumin/creatinine ratio.
