# Supplementary material for: Alterations in Monocyte CD16 in Association with Diabetes Complications
Source: Mediators Inflamm. 2012 Dec 18;2012:649083. doi: 10.1155/2012/649083 (PMC3536440; doi:10.1155/2012/649083)
Supplement: Supplementary file 1 — Supplemental Table 1: listed the details regarding individual complications status of all diabetic subjects in this study [file 649083.f1.pdf]

Supplementary Table 1. Individual diabetic subject's complications status.

Study Code	Age (years)	Duration (years)	Macrovascular	Microvascular	
				Nephropathy	Retinopathy
**5**	61.9	15.3	-	-	+
**7**	57.8	44.3	-	-	+
**8**	30.7	30.7	-	+	+
**9**	73.2	24.4	-	+	+
**12**	62.4	13.4	-	+	+
**14**	72.7	13.5	+	+	+
**15**	54.5	17.5	-	-	+
**16**	59.8	16.5	+	+	+
**17**	78.8	13.5	-	+	+
**18**	58.0	11.5	-	+	+
**19**	76.4	26.7	-	+	+
**20**	35.6	24.7	-	+	+
**21**	58.8	18.7	+	-	+
**22**	66.3	26.8	-	+	+
**23**	35.0	23.8	-	-	+
**24**	77.4	23.8	-	+	+
**27**	75.5	20.0	-	+	+
**28**	78.4	27.8	+	+	+
**1**	57.2	12.1	-	-	-
**2**	71.2	20.7	-	-	-
**3**	68.7	11.1	-	-	-
**4**	70.0	22.1	-	-	-
**6**	46.9	19.3	-	-	-
**10**	54.2	13.3	-	-	-
**11**	71.4	12.3	-	-	-
**13**	47.9	23.4	-	-	-
**25**	65.0	15.0	-	-	-
**26**	62.3	11.3	-	-	-
**29**	57.5	39.9	-	-	-
**30**	62.1	12.6	-	-	-
**31**	55.1	13.9	-	-	-
**32**	67.7	10.5	-	-	-
**33**	47.9	28.2	-	-	-
**34**	67.7	18.4	-	-	-
**35**	70.5	22.2	-	-	-
**36**	76.7	20.4	-	-	-
**37**	64.8	14.4	-	-	-
**38**	62.7	19.6	-	-	-
**39**	69.4	12.6	-	-	-
**40**	58.2	23.6	-	-	-
**41**	77.3	13.6	-	-	-
**42**	70.4	20.6	-	-	-
**43**	59.4	13.6	-	-	-
